# The Canadian Medical Association

**Published:** 1876-08

**Authors:** 


### The Canadian Medical Association.

We had the pleasure of attending the Ninth Annual Meeting of the Canadian Medical Association, held at Toronto, August 2, 1876, and met with a hearty reception from our Canadian brethren.

The papers read before the Association all evinced a high order of intelligence among the members, and were ably discussed by those present.

The President, Dr. Hodder of Toronto, welcomed the medical men present from the United States in a hearty manner; he alluded to thework which was being done by the Medical Societies upon this side and held their example up as a stimulant to the energies of the Canadian Association After a few remarks of an introductory character in his address, he dwelt upon the treatment of fibroid tumors of the uterus and somewhat upon the operation of ovaritomy. He alluded to the recent case operated upon by Prof. Thomas of New York, in which the transfusion of milk was employed to sustain the patient, an operation first made by Dr. Hodder, several years ago in a cholera epidemic.

Dr. Joseph Workman, of Toronto, read to the Association a long and interesting address upon the relation of Crime and Insanity. His paper was one of great interest and was written in a strong and able manner. He laid at the door of the press a large share of responsibility for the large number of suicides which are daily occurring. He said that the great demand for something sensational in character for the pages of the daily press led to the publication of the particulars of great crimes and notable suicides, in all their horrible details, which only served to awaken the faculty of imitation which is so strong a feature of many weak minds, leading to increased crime and suicide. He cited instances where the first impulse to commit crime was awakened in the mind of the murderer by reading or hearing of some similar occurrence. The paper was a powerful plea against the present sensational style of writing for the public press.

The discussion upon Dr. Workman's paper was followed by a paper by by Dr. Strange, of Aurora, Ont., upon the treatment of the Pedicle in Ovariotomy. We hope to publish this paper in full shortly. In the evening a paper was read by Dr. Rosebrugh upon the Physiology of Menstruation, following which was an extended discussion upon Vital Statistics and Public Hygiene. We were unable to be present at the second day's meeting, but we understand that it was fully equalled in that of the first. Our Canadian brethren have inaugurated a good work in forming an Association, and while they labor under a disadvantage of having and interior not yet developed and supplied with convenient lines of railway, to bring them together, thus being obliged to draw from a frontier extending from Halifax to the lakes, they made up what is lacking in quantity by the excellent quality of their meetings.

The next meeting of the Association will be held in Montreal the second Wednesday in September, Dr. Hingston, of Montreal, President.

